# Prognostic impact of radiotherapy-induced-lymphopenia in patients treated with breast-conservative surgery

**DOI:** 10.1038/s41598-023-41301-3

**Published:** 2023-09-01

**Authors:** Chang Ik Yoon, Jawon Hwang, Dooreh Kim, Jung Hwan Ji, Janghee Lee, Soong June Bae, Joon Jeong, Jee-Suk Chang, Yeona Cho, Hye Sun Lee, Jee Ye Kim, Sung Gwe Ahn

**Affiliations:** 1 Department of Surgery, College of Medicine, Seoul St Mary’s Hospital, The Catholic University of Seoul, Seoul, Republic of Korea; 2 Department of Surgery, Gangnam Severance Hospital, Yonsei University College of Medicine, 211 Eonju-ro, Dogok 1(il)-dong, Gangnam-gu, Seoul, 06273 Republic of Korea; 3 Institute for Breast Cancer Precision Medicine, Yonsei University College of Medicine, Seoul, Republic of Korea; 4 Department of Surgery, Hallym University Dongtan Sacred Heart Hospital, Hwaseong, Republic of Korea; 5 Department of Radiation Oncology, Gangnam Severance Hospital, Yonsei University College of Medicine, Seoul, Republic of Korea; 6 Biostatistics Collaboration Unit, Yonsei University College of Medicine, Seoul, Republic of Korea; 7 Division of Breast Surgery, Department of Surgery, Breast Cancer Center, Yonsei Cancer Center, Severance Hospital, Yonsei University College of Medicine, 50-1 Yonsei-ro, Sinchon-dong, Seodaemun-gu, Seoul, 03722 Republic of Korea

**Keywords:** Cancer, Oncology

## Abstract

We investigated a prognostic impact of radiotherapy-induced lymphopenia (RIL) in breast cancer patients treated with breast-conservative surgery (BCS). We included 531 breast cancer patients who were treated with BCS and adjuvant radiotherapy. None of these received (neo)adjuvant chemotherapy. Pre- and post- absolute lymphocyte counts (ALC) were reviewed before and after radiotherapy. The primary endpoint was to evaluate recurrence-free survival (RFS) according to the pre-to-post ALC ratio. Binary logistic regression model was used to identify risk factors for RIL. Either continuous or categorical (> 2.4) pre-to-post ALC ratio was associated with RFS. In 531 patients receiving whole breast irradiation (WBI) and regional nodal irradiation (RNI), RFS was significantly reduced in the patients with high pre-to-post ALC ration (> 2.4). In multivariable analysis, low pre-to-post post ALC ratio was significantly related to decreased RFS in the multivariable analysis (HR 2.293, 95% CIs 1.110–4.735, P = 0.025). In 452 patients treated with WBI alone, high pre-to-post ALC ratio was still significantly associated with decreased RFS in the multivariable analysis (HR 2.708, 95% CIs 1.016–7.218, P = 0.046). In binary logistic regression analysis, RNI was only significant risk factor for clinically meaningful RIL. Our findings show that a markedly decrease in ALC during radiotherapy has a negative prognostic impact.

## Introduction

Radiation therapy (RT) reduces locoregional recurrence and distant metastasis in breast cancer^1^. Breast irradiation after breast-conserving surgery (BCS), in particular, is an integral part of breast-conservative treatment and has been a standard of care for early breast cancer, as evidenced by long-term cumulative survival data^2^.

Localized irradiation may have a deleterious effect on host immunity, particularly affecting counts of lymphocytes and their subpopulations^3^. RT-induced lymphopenia (RIL) has been linked to poor survival outcomes in patients with various solid cancers, such as lung cancer, hepatocellular carcinoma, glioma, and breast cancer^4–6^. Specifically, in patients treated with breast-conserving surgery followed by RT, it is reported that post-RT lymphopenia could be a potential risk factor for ipsilateral breast tumor recurrence^7^. In addition, among the patients receiving post-mastectomy RT, a low nadir- absolute lymphocyte count (ALC)/pre-ALC ratio (< 0.8) was associated with poor prognosis^8^. Another study reported that the minimum ALC after cancer treatment is associated with overall survival in patients with triple-negative breast cancer (TNBC)^9^. Thus, it is important to identify risk factors for RIL after irradiation in patients with breast cancer.

In this study, we investigated ALC before and after RT in breast cancer patients who received whole breast irradiation (WBI) after BCS but were not treated with (neo)adjuvant chemotherapy. Based on a pre-to-post ALC ratio, which could assess a magnitude of reduced ALC by RT independent of pre-RT or post-RT ALC, we investigated a prognostic impact of RIL. In addition, we sought to identify risk factors including body mass index (BMI) for a high pre-to-post ALC ratio, which reflects RIL.

## Results

### Study population

**Figure 1:**
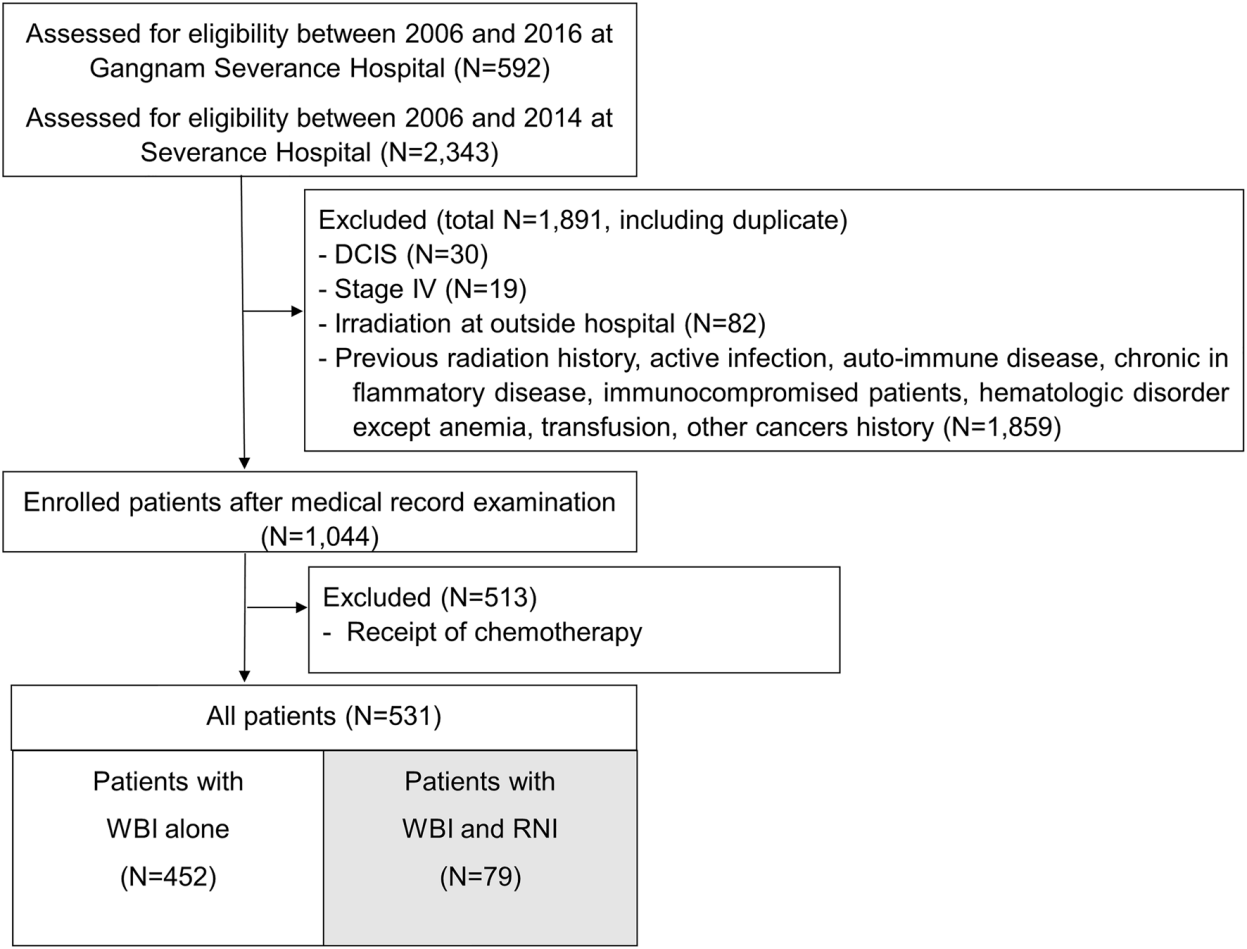
Patients flow diagram for the selection and enrollment of eligible patients in this study. WBI: whole breast irradiation; RNI: regional nodal irradiation.

A total of 2935 patients with breast cancer were assessed at Gangnam Severance Hospital and Severance Hospital (Fig. 1). We excluded patients with ductal carcinoma in situ (n = 30), de novo stage IV (n = 19), irradiation outside the hospital (n = 82), previous medical history such as infection, previous irradiation history, autoimmune disease, inflammatory disease, immunocompromised state, hematologic disorders except anemia, transfusion history, and other malignancies except thyroid cancer (n = 1859). Among 1044 patients who met the inclusion criteria, 531 who did not undergo chemotherapy were included in the anaylses (Fig. 1). Of these, 452 (85.1%) received WBI alone, whereas 79 (14.9%) received WBI and regional nodal irradiation (RNI) (Fig. 1). Baseline demographics of the patients are presented in Table 1.

**Table 1:** Baseline characteristics of breast cancer patients who underwent radiotherapy after breast conserving surgery.

	Total (n = 531)
Age (year, continuous)	52.57 ± 9.81
BMI (kg/m^2^, continuous)	23.20 ± 3.19
Pre-ALC (cells/μL)	1867.65 ± 1019.66
Post-ALC (cells/μL)	571.82 ± 357.54
ER	
Positive	459 (86.4)
Negative	72 (13.6)
PR	
Positive	368 (69.3)
Negative	161 (30.3)
Missing	2 (0.4)
HER2	
Positive	166 (31.3)
Negative	360 (67.8)
Missing	5 (0.9)
HG	
I, II	434 (81.7)
III	62 (11.7)
Missing	35 (6.6)
Subtype	
Luminal/HER2(−)	318 (59.9)
HER2 (+)	165 (31.1)
TNBC	41 (7.7)
Missing	7 (1.3)
Stage	
I	464 (87.4)
II	55 (10.4)
III	6 (1.1)
Missing	6 (1.1)
Tumor size	
≤ 2 cm	496 (93.4)
> 2 cm	32 (6.0)
Missing	3 (0.6)
Node metastasis	
No	495 (93.2)
Yes	34 (6.4)
Missing	2 (0.4)
Endocrine treatment	
Done	476 (89.6)
Not done	55 (10.4)
RT dose (cGy, continuous)	5914.37 ± 255.73 (n = 530)
RNI	
Done	452 (85.1)
Not done	79 (14.9)

ALC, absolute lymphocyte count; BMI, body mass index; ER, estrogen receptor; PR, progesterone receptor; HER2, human epidermal growth factor receptor 2; HG, histologic grade; TNBC, triple negative breast cancer; RNI, regional nodal irradiation.

### Survival based on the pre-to-post ALC ratio in all patients

At a median follow-up period of 96 months (range: 2–151 months), 33 patients had recurrences. Among them, 29 had distant metastasis, 8 had locoregional recurrence, and 4 had distant and loco-regional recurrence simutaneously. There were 16 deaths. Continuous pre-to-post ALC ratio was a significant prognostic factor for recurrence-free survival (RFS). In the multivariable model, continuous pre-to-post ALC ratio was demonstrated to be a signficant prognostic factor independent of estrogen receptor (ER), progesteribe receotir (PR), human epidermal growth factor receptor 2 (HER2), histologic grade (HG), and stage (Supplementary Table 1).

To optimize the cut-off point, we used time-dependent receiver operating characteristic (ROC) curve. The area under the ROC curve was 0.625 [Supplementary Fig. 1; 95% confidence intervals (CIs): 0.512–0.738, p = 0.0161]. The obtained cut-off value was 2.4 based on the Youden’s index.

A total of 531 patients were categorized based on low and high pre-to-post ALC ratios (cut-off point: 2.4). Baseline demographics of two groups divided by the ratio are presented in Supplementary Table 2. The high pre-to-post ALC group had a higher stage, more frequent nodal metastasis, a higher BMI, an aggressive breast cancer subtype, and had received RNI.

**Figure 2:**
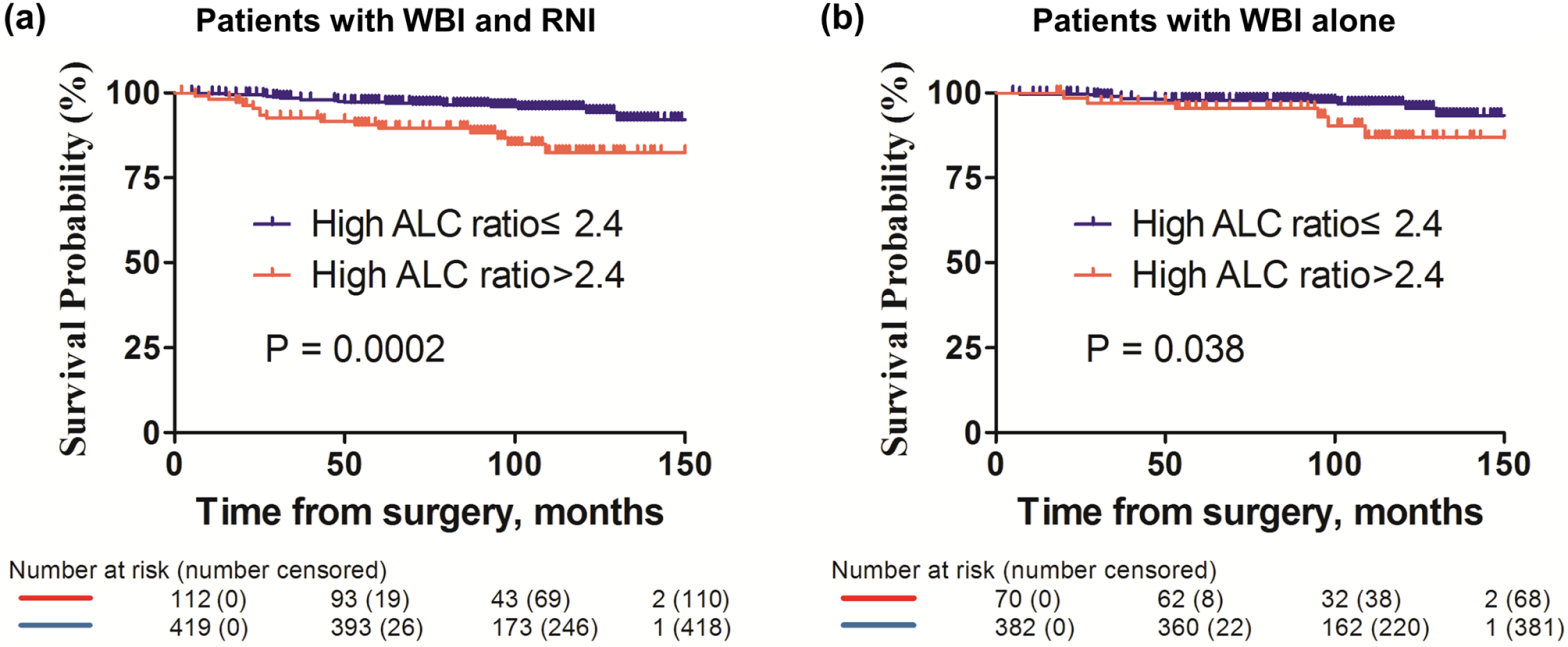
Kaplan–Meier survival curves of RFS according to the pre-to-post ALC ratio. (**a**) Kaplan–Meier survival curve of RFS based on the pre-to-post ALC ratio. Patients with high pre-to-post ALC ratios exhibitied poor RFS (**a**, p = 0.0002). (**b**) Kaplan–Meier survival curve of RFS based on the pre-to-post ALC ratios in patients withour receiving RNI. Patients with high pre-to-post ALC ratios exhibitied poor RFS (**b**, p = 0.038). RFS: recurrence-free survival; ALC: absolute lymphocyte count; WBI: whole breast irradiation; RNI: regional nodal irradiation.

In survival analysis with the dichotomized ratio, patients with a high pre-to-post ALC ratio (> 2.4) were significantly associated with decreased RFS (Fig. 2a; p = 0.0002, log-rank test). In the univariable Cox proportional hazard model, pre-to-post ALC ratio [Supplementary Table 3, hazard ratio (HR): 3.375, 95% CIs: 1.701–6.698, p = 0.001], ER status, PR status, HER2 status, HG, and stage were found to be significant prognostic factors for RFS. In multivariable model with significant factors selected by univariable analyses, a high pre-to-post ALC ratio was significantly associated with decreased RFS (Supplementary Table 3, HR: 2.380, 95% CI 1.146–4.942, p = 0.020).

### Survival in patients with WBI alone

Since a part of the study population were treated with additional RNI, which might have aggravated lymphocyte depletion, we further analyzed in the patients treated with WBI alone. A total of 452 patients were included and classified into two groups according to the pre-to-post ALC ratio (Fig. 1). Baseline demographics of the patients are presented in Table 2. There was no statistically significant difference in baseline demographics including pathological factors and total radiation dose between the two groups. In survival analysis, RFS differed significantly by the pre-to-post ALC ratio (Fig. 2b, p = 0.038, log-rank test).

**Table 2:** Clinical characteristics according to pre-to-post ALC ratio in patients with WBI alone.

	ALC ratio > 2.4, n = 70 (%)	ALC ratio ≤ 2.4, n = 382 (%)	*P* value
Age (year, continuous)	53.13 ± 10.21	51.76 ± 9.68	0.280
BMI (kg/m^2^, continuous)	23.67 ± 3.34	23.06 ± 3.11	0.134
Pre-ALC (cells/μL)	2236.04 ± 574.97	1802.13 ± 543.74	< 0.001
Post-ALC (cells/μL)	770.51 ± 222.26	1123.76 ± 336.80	< 0.001
ER			0.472
Positive	59 (84.3)	334 (87.4)	
Negative	11 (15.7)	51 (12.6)	
PR			0.171
Positive	49 (70.0)	273 (71.5)	
Negative	21 (30.0)	107 (28.0)	
Missing	0	2 (0.5)	
HER2			0.074
Positive	15 (21.4)	123 (32.2)	
Negative	54 (77.1)	255 (66.8)	
Missing	1 (1.4)	4 (1.0)	
HG			0.647
I, II	56 (80.0)	313 (81.9)	
III	9 (12.9)	42 (11.0)	
Missing	5 (7.1)	27 (7.1)	
Subtype			0.055
Luminal/HER2(−)	45 (64.3)	230 (60.2)	
HER2 (+)	15 (21.4)	122 (31.9)	
TNBC	9 (12.9)	24 (6.3)	
Missing	1 (1.4)	6 (1.6)	
Stage			0.474
I	63 (90.0)	354 (92.7)	
II	6 (8.6)	24 (6.3)	
Missing	1 (1.4)	4 (1.0)	
Tumor size			0.768
≤ 2 cm	66 (94.3)	363 (95.0)	
> 2 cm	4 (5.7)	19 (5.0)	
Node metastasis			0.052
No	65 (92.9)	375 (98.2)	
Yes	4 (5.7)	6 (1.6)	
Missing	1 (1.4)	1 (0.3)	
Endocrine treatment			0.503
Done	62 (88.6)	348 (91.1)	
Not done	8 (11.4)	34 (8.9)	
RT dose (cGy, continuous)	5951.04 ± 64.13	5906.42 ± 293.95	0.217

BMI, body mass index; ALC, absolute lymphocyte count; ER, estrogen receptor; PR, progesterone receptor; HER2, human epidermal growth factor receptor 2; HG, histologic grade; TNBC, triple negative breast cancer; RT, radiotherapy.

In the univariable Cox proportional hazard model, pre-to-post ALC ratio (Table 3, HR 2.704, 95% CIs 1.014–7.206, p = 0.047), and stage were found to be significant factors for recurrence. A high pre-to-post ALC ratio was demonstrated to be a significant factor for decreased RFS in the multivariable analysis (Table 3, HR 2.708, 95% CIs 1.016–7.218, p = 0.046).

**Table 3:** Hazard ratios (HRs) and 95% confidential intervals (CIs) for recurrence-free survival (RFS) in patients with WBI alone.

	Univariate analysis		Multivariate analysis	
	HR (95% CIs)	*P* value	HR (95% CIs)	*P* value
Age	0.981 (0.931–1.034)	0.474		
BMI	0.961 (0.815–1.134)	0.641		
Pre-to-post ALC ratio		0.047		0.046
≤ 2.4	1		1	
> 2.4	2.704 (1.014–7.206)		2.708 (1.016–7.218)	
ER		0.252		
Negative	1			
Positive	0.522(0.172–1.588)			
PR		0.489		
Negative	1			
Positive	0.707 (0.265–1.887)			
HER2		0.568		
Negative	1			
Positive	1.333 (0.497–3.574)			
Subtype				
Luminal/HER2(−)	1			
HER2 (+)	1.590 (0.563–4.490)	0.381		
TNBC	2.775 (0.750–10.257)	0.126		
HG		0.115		
I, II	1			
III	2.319 (0.815–6.595)			
Stage		0.049		0.133
I	1		1	
II	3.466 (1.001–12.009)		2.695 (0.738–9.841)	

HR, hazard ratio; 95%CIs, 95% confidence intervals; BMI, body mass index; ALC, absolute lymphocyte count; ER, estrogen receptor; PR, progesterone receptor; HER2, human epidermal growth factor receptor 2; HG, histologic grade.

### Baseline BMI and risk factors for a high pre-to-post ALC ratio

**Figure 3:**
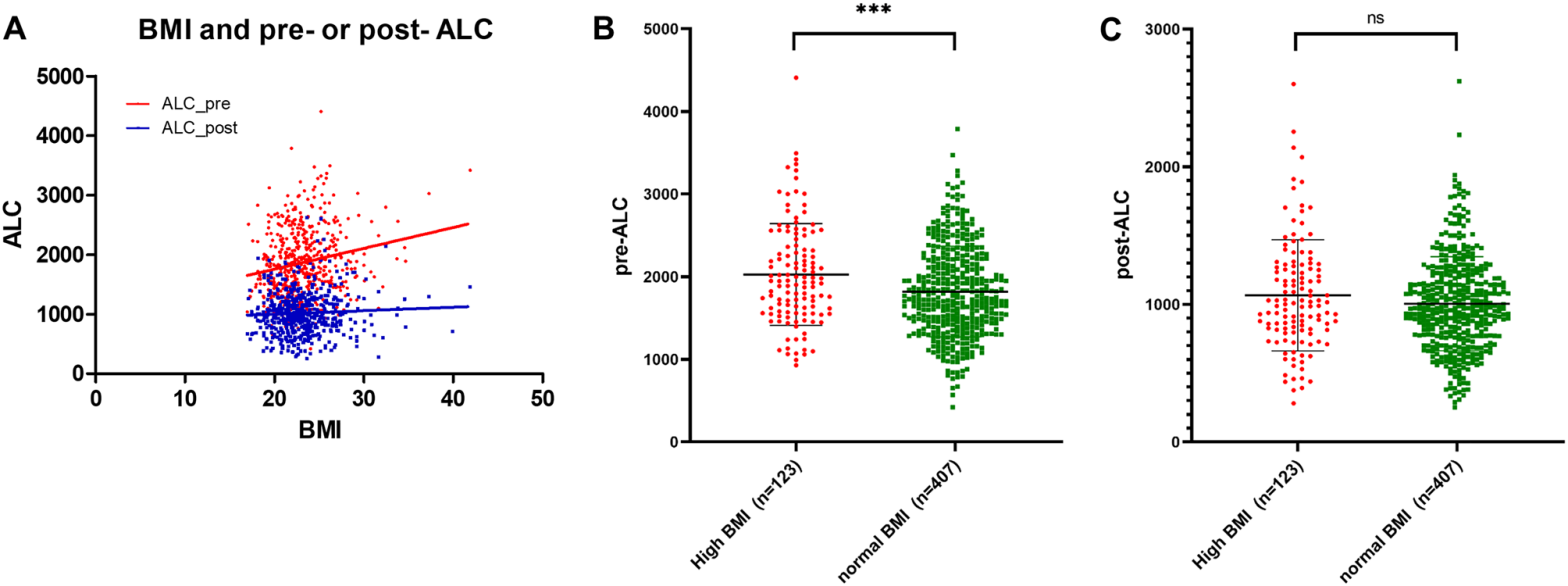
Pearson correlation analysis between BMI and ALC, and comparison of pre- and post-ALC between the high BMI group and normal BMI group. (**A**) Pearson correlation analysis between BMI and ALC. The correlative value between pre-ALC and BMI (Pearson’s r) was 0.195 (p < 0.001, red line and red dot). However, there was no correlation between post-ALC and BMI in all patients (Pearson’s r = 0.051, p = 0.238). Comparisons of pre- (**B**) and post-ALC (**C**) between the two groups stratified by BMI. The obese or overweight patients had a signficantly higher mean pre-ALC than non-obese patients (Fig. 1B, BMI ≥ 25, pre-ALC: 2027.51 ± 616.18; BMI < 25, pre-ALC: 1820.18 ± 549.97, p < 0.001). However, there was no difference in the mean post-ALC between the two groups (Fig. 1C, BMI ≥ 25, post-ALC: 1066.65 ± 403.99; BMI < 25, post-ALC: 1005.53 ± 342.00, p = 0.097). BMI: body mass index; ALC: absolute lymphocyte count.

Since previous studies showed that BMI is correlated with ALC, we investiaged the relationship between BMI and pre- or post-RT ALC. Baseline BMI was correlated with pre-ALC before irradiation (Fig. 3A, Pearson’s r = 0.195, p < 0.0001). However, it was not corrleated with post-ALC close to the last session of RT (Fig. 3A, Pearson’s r = 0.051, p = 0.238). When we compared the mean values of pre-ALC based on BMI, obese or overweight patients had a signficantly higher mean pre-ALC than non-obsese patients (Fig. 3B, p < 0.001). However, there was no difference in the mean values of post-ALC between the two groups (Fig. 3C, p = 0.097).

**Figure 4:**
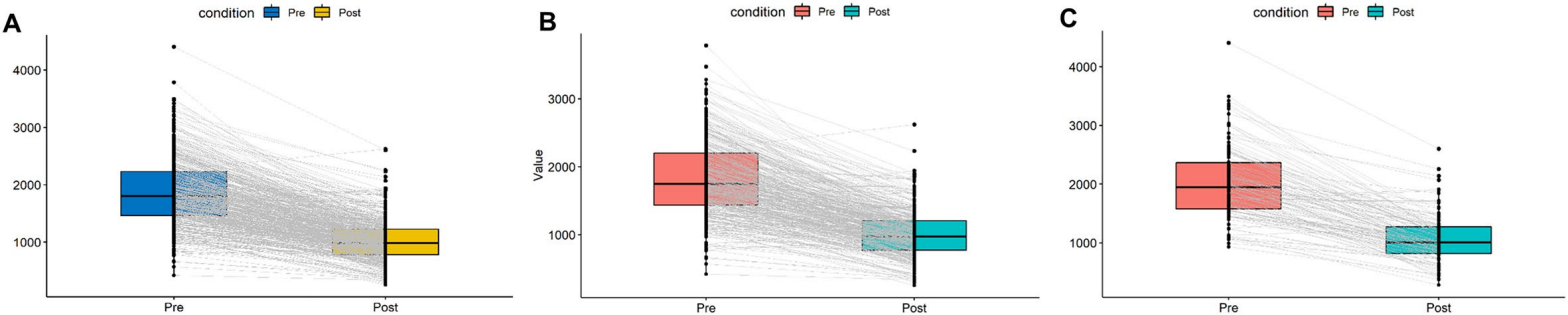
Comparison of ALC change pre-and post-irradiation. (**A**) The mean values of post-ALC were signifianctly lower than those of pre-ALC in the both groups (p < 0.001, paired t-test). (**B**) In the high-BMI group (p < 0.001, paired t-test). (**C**) In the normal-BMI group (p < 0.001, paired t-test). ALC: absolute lymphocyte count; BMI: body mass index.

The change in ALC pre- and post-irradiation in all patients is shown in Fig. 4A. When compared to pre-ALC, post-ALC was reduced by a mean difference of 848.00 ± 520.65 (Fig. 4A, p < 0.001, paired t-test). In normal BMI patients, the difference in ALC before and after irradiation was 814.65 ± 510.84 (Fig. 4B, p < 0.001), and in high BMI patients, the difference in ALC was 960.86 ± 540.25 (Fig. 4C, p < 0.001).

Next, the clinical and pathological factors associated with a high pre-to-post ALC ratio (> 2.4) were analyzed by binary logistic regression analysis (Table 4). In univariable analysis, continuous BMI, TNBC subtype, stage, node metastasis, and RNI were found to be associated with a high pre-to-post ALC ratio (> 2.4), indicating clinically meaningful RIL. In the multivariable model, only RNI (HR 5.586, 95% CIs 3.067–10.174, p < 0.001) were significant risk factors for RIL.

**Table 4:** Predicting clinic-pathologic factors for RIL using binary logistic regression analysis.

	Univariable analysis		Multivariable analysis	
	OR (95% CIs)	*P* value	OR (95% CIs)	*P* value
Age (year, continuous)	1.008 (0.987–1.030)	0.433		
BMI (kg/m^2^, continuous)	1.065 (1.011–1.121)	0.048	1.052 (0.978–1.131)	0.173
ER		0.073		
Negative	1			
Positive	0.601 (0.344–1.049)			
PR		0.172		
Negative	1			
Positive	0.736 (0.474–1.143)			
HER2		0.167		
Negative	1			
Positive	0.718 (0.448–1.149)			
HG		0.108		
I,II	1			
III	1.632 (0.899–2.963)			
Subtype				
Luminal/HER2(−)	1		1	
HER2 (+)	0.799 (0.493–1.295)	0.362	0.768 (0.448–1.316)	0.336
TNBC	2.161 (1.084–4.310)	0.029	1.882 (0.813–4.354)	0.140
Stage				
I	1		1	
II	2.973 (1.650–5.354)	< 0.001	0.841 (0.305–2.318)	0.737
III	4.459 (0.885–22.475)	0.070	0.456 (0.055–3.757)	0.466
Tumor size		0.317		
≤ 2 cm	1			
> 2 cm	1.504 (0.676–3.350)			
Node metastasis		< 0.001		0.225
No	1		1	
Yes	5.549 (2.717–11.329)		2.260 (0.605–8.444)	
RNI		< 0.001		< 0.001
Done	1		1	
Not done	6.195 (3.719–10.317)		5.586 (3.067–10.174)	
Endocrine treatment		0.062		
Done	1			
Not done	1.794 (0.971–3.317)			

RIL, radiation-induced lymphopenia; OR, odd ratio; 95%CIs, 95% confidence intervals; BMI, body mass index; ER, estrogen receptor; PR, progesterone receptor; HER2, human epidermal growth factor receptor 2; HG, histologic grade; TNBC, triple negative breast cancer; RNI, regional nodal irradiation.

## Discussion

In this study, we found that lymphocytes were depleted after RT and demonstrated that patients with marked lymphocyte depletion after RT showed an inferior RFS than those with mild lymphocyte depletion. Risk factors for considerable RIL were node-positivity and RNI.

We found that substantial RIL was clearly associated with poor RFS in patients treated without chemotherapy. We believe that this is the first study to investigate survival outcome with pre-to-post ALC ratio in breast cancer patients treated with irradiation after BCS. Higher pre-to-post ALC ratios were found to be significantly related to a higher recurrence rate than lower pre-to-post ALC ratios. These findings indicated that pre-to-post ALC ratio could be utilized as a biomarker reflecting host immunity resistance to RT in breast cancer patients.

In our study, BMI was not associated with post-RT ALC and did not attenuate a harmful effect of RT on lymphocytes. As mentioned in the introduction, previous studies showed that ALC generally correlates with BMI^10^ and provided clinical evidence that low BMI prior to RT would be a risk factor for RIL^8, 11–13^. Thus, we hypothesized that high BMI could reduce RIL in patients who were solely treated with RT. However, our findings indicate that a high BMI does not protect against the harmful effects of RT on lymphocytes, even though BMI was correlated with ALC at baseline.

Lymphocytes, which include T cells, B cells, and natural killer cells, account for 20–44% of circulating white blood cells. It is well known that lymphocytes are the most radiosensitive cells among the bone marrow cells, including the erythroid, myeloid, and lymphoid lineage. Yovino et al. have demonstrated the impact of RT on reducing lymphocyte count using a mathematical model to calculate the radiation dose to circulating lymphocytes in patients receiving irradiation^14^. In irradiated patients, the decrease in lymphocyte count persisted for more than two years and was reduced by up to 60%^15^. Upadhyay et al. also demonstrated through a meta-analysis that advanced age, lower baseline lymphocyte count, larger tumor size, and advanced stage in lung cancer were the risk factors for RIL^16^. With this background, lymphocyte-sparing RT or hypofractional RT is highlighted to maintain more lymphocytes alive^8, 17, 18^. In fact, we also found that nodal metastasis and RNI were risk factors for profound RIL. We added clinical evidence supporting the previous findings that a larger field of RT affects adversely lymphocyte counts.

This study has several limitations. First, we tested complete blood count (CBC) to obtain post-ALC before the completion of RT. Thus, almost all patients were in a state of lymphocyte depletion. In addition, due to the retrospective design of the study, the timing of blood sampling was not uniform. This peripheral blood sampling time should be considered when addressing our findings. Another limitation is that we cannot address radiation dosimetry factors such as lung, heart dose, and the extent of the radiation field including mediastinum and great vessels due to the retrospective study design. Pre-planned study to examine an effect of RT-dosimetry factors on RIL is warranted.

Furthermore, the cut-off value of pre-to-post ALC ratio should be validated in future studies. If we exchanged our ratio to a post-to-pre ALC ratio, the cut-off was 0.42. It was different compared to the study by Sun et al.^8^. Since there are several differences between two studies. Their patients were treated with post mastectomy radiation therapy, whereas our patients were treated with mainly WBI after BCS. In addition, they used the nadir post-ALC and collected blood sampling 1 month after the end of radiation, not during RT, suggesting that ALC could be recovered partly. By contrast, we used post-ALC before the completion of RT, and almost all patients were in a state of lymphocyte depletion.

Despite these limitations, using a cohort consisting of relatively homogenous patients who underwent RT after BCS and were not treated with chemotherapy, we identified that a marked decrease in ALC during the course of RT has a negative prognostic impact. In addition, an increasing BMI is related to a high pre-to-post ALC ratio, necessitating further research on the association between obesity and susceptibility of lymphocytes to RT. A multidisciplinary approach, including radiation techniques, should be considered to reduce RIL and preserve host immunity during RT.

## Methods and materials

### Study population

We retrospectively collected data from patients who underwent curative resection for primary breast cancer between January 2006 and December 2016 at Gangnam Severance Hospital and between January 2006 and December 2014 at Severance Hospital. The patients’ clinicopathologic information was extracted from electrical medical records. Patients older than 19 years who were diagnosed with histologically confirmed invasive breast carcinoma in stages I–III and underwent BCS followed by radiation treatment were enrolled. To circumvent the influence of chemotherapy on lymphopenia, we only included patients who had not undergone (neo)adjuvant chemotherapy. The exclusion criteria were diseases with only in situ lesions or distant metastases and patients with active infection, inflammatory disease, autoimmune disease, immunocompromised state, hematologic disorders except anemia, a history of previous irradiation, and history of blood transfusions that could be identified in medical records.

The following clinicopathological data were collected: age, weight, height, HG, tumor size, lymph node metastasis, hormone receptor status (ER and PR), HER2, receipt of endocrine therapy, and CBC. TNM staging was performed in accordance with the7^th^ edition of the American Joint Committee on Cancer, and tumor grade was defined using the modified Scarff-Bloom-Richardson grading system^19^.

This study was performed in accordance with the Good Clinical Practice guidelines and the principles of the Declaration of Helsinki and was approved by the Institutional Review Board (IRB) at Gangnam Severance Hospital, Yonsei University, Seoul, Republic of Korea (IRB number: 3-2018-0341). The requirement for informed consent was waived due to the retrospective study design.

### BMI, pre- and post-ALC, and radiation protocol

Body weight and height of the enrolled patients were measured on their first visit or on admission for breast operation. All measurements were taken prior to any treatment for breast cancer. BMI was calculated by dividing body weight (kilogram) by the square of height (meter), as defined by the World Health Organization^20^. When we used categorical BMI, we divided the enrolled patients into two groups based on BMI: (i) high (BMI ≥ 25.0 kg/m^2^) and (ii) normal weight (BMI < 25.0 kg/m^2^)^20^.

Peripheral blood samplings were taken at baseline, two weeks prior to RT (pre-ALC) and one week before the last RT lesion (post-ALC). White blood cells and differential counts were evaluated at the Department of Laboratory Medicine using an automated counting machine (Sysmex XN-Series; Kobe, Japan). The pre- and post-ALCs were determined using differential counts, and the pre-to-post ALC ratio was obtained.

Radiation was administered according to the following protocol: 50.4 Gy of radiation was administered in 28 fractions using X-ray linear accelerators (Elekta; Stockholm, Sweden) to the whole breast, followed by a boost dose of 9 Gy in 5 fractions delivered to the tumor bed. A few patients were treated with hypofractionated RT (5/532, 0.9%). In cases with positive axillary lymph nodes or suspicious internal mammary lymph nodes, RNI was delivered simultaneously.

### Statistical analysis

The primary end point was RFS. Recurrence was defined as any type of recurrence, including locoregional recurrence and distant metastasis. RFS was calculated from the date of primary breast surgery to the censored date. Survival curves were drawn from the Kaplan–Meier estimator and compared between the two groups using the log-rank test. We plotted ROC curves to determine the cut-off value of the pre-to-post ALC ratio in predicting risk of recurrence. The cut-off value was determined by the maximum sum of sensitivity and specificity. To identify independent variables for RFS, we used multivariable Cox proportional hazard models.

The Pearson’s Correlation coefficient was calculated to measure the correlative value between continuous BMI and ALC. Descriptive statistics were used to demonstrate the distribution of all covariates between the two groups based on pre-to-post ALC ratio. Differences in baseline characteristics were compared using the Student’s *t*-test for continuous variables and the chi-square test for categorical variables. Mean values of the pre- and post-ALC were compared using the paired *t*-tests.

To identify risk factors for RIL, the binary logistic regression model was used. The variables used in the multivariate analysis were those that showed statistical significance in the univariable analysis. Statistical analysis was conducted using SPSS version 24 (SPSS; Chicago, IL, USA), SAS version 9.4 (SAS Institute, Cary, NC, USA) and the R software (https://www.r-projet.org; version 3.6.1). Statistical significance was defined as a p-value less than 0.05 and a 95% confidence interval (CI) excluding 1.

### Ethics approval and consent to participate

All procedures performed in studies involving human participants were in accordance with the ethical standards of the institutional and/or national research committee and with the 1964 Declaration of Helsinki and its later amendments or comparable ethical standards. The protocol was approved by the Institutional Review Board (Local IRB number: 3-2018-0341) of Gangnam Severance Hospital. The need for informed consent was waived by the IRB due to the retrospective study design.

### Supplementary Information

Supplementary Figure 1.

Supplementary Tables.

## Data Availability

All data generated or analyzed during this study are included in this research article and the supplementary information files.

## Abbreviations

RT: Radiation therapy
BCS: Breast-conserving surgery
RIL: RT-induced lymphopenia
ALC: Absolute lymphocyte count
TNBC: Triple-negative breast cancer
WBI: Whole breast irradiation
BMI: Body mass index
RNI: Regional nodal irradiation
RFS: Recurrence-free survival
ER: Estrogen receptor
PR: Progesterone receptor
HER2: Human epidermal growth factor receptor 2
HG: Histologic grade
ROC: Receiver operator characteristic
CI: Confidence interval
HR: Hazard ratio
CBC: Complete blood count
IRB: Institutional Review Board
