# Supplementary figures and images for: Prognostic impact of radiotherapy-induced-lymphopenia in patients treated with breast-conservative surgery
Source: Sci Rep. 2023 Sep 1;13:14372. doi: 10.1038/s41598-023-41301-3 (PMC10474014; doi:10.1038/s41598-023-41301-3)

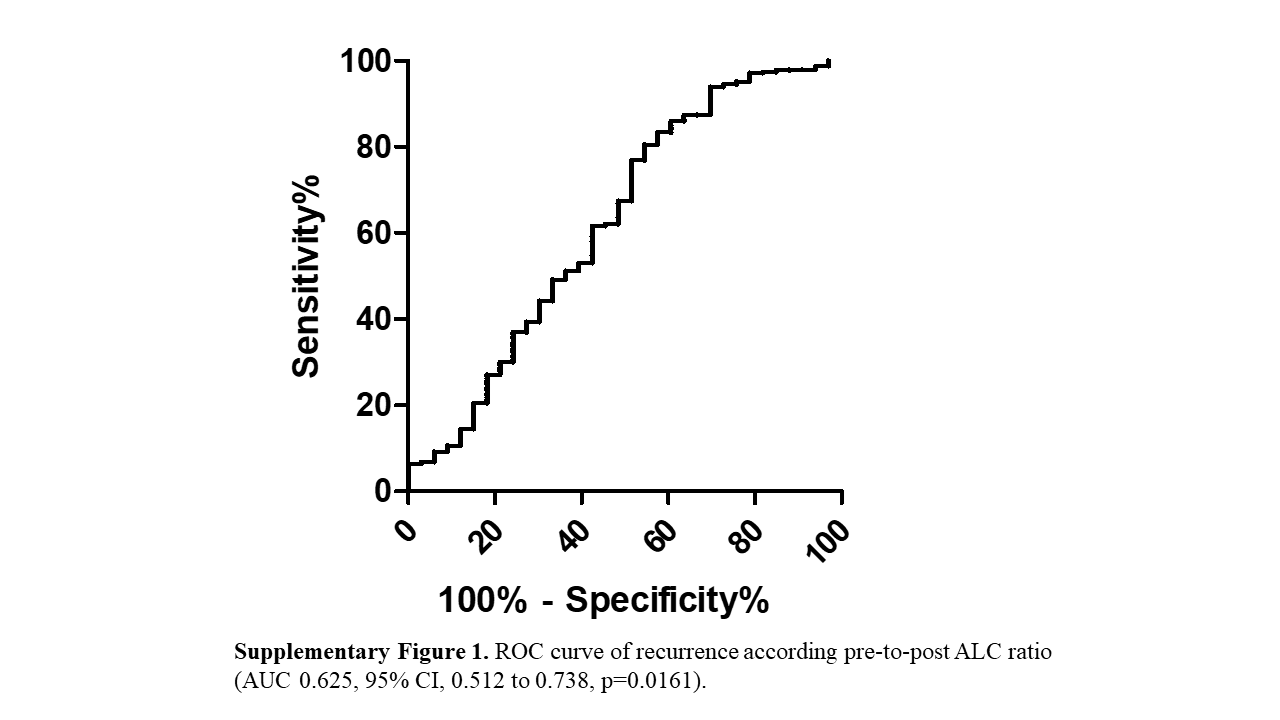

Supplement: Supplementary file 1 — Supplementary Figure 1. [file 41598_2023_41301_MOESM1_ESM.tif]
