# Supplementary material for: Prognostic impact of radiotherapy-induced-lymphopenia in patients treated with breast-conservative surgery
Source: Sci Rep. 2023 Sep 1;13:14372. doi: 10.1038/s41598-023-41301-3 (PMC10474014; doi:10.1038/s41598-023-41301-3)
Supplement: Supplementary file 2 — Supplementary Tables. [file 41598_2023_41301_MOESM2_ESM.docx]

**Supplementary Table 1. Hazard ratios (HRs) and 95% confidential intervals (CIs) for recurrence-free survival (RFS)**

|  | **Univariate analysis** | | **Multivariate analysis** | |
| --- | --- | --- | --- | --- |
|  | **HR (95% CIs)** | ***P* value** | **HR (95% CIs)** | ***P* value** |
| **Age** | 0.991 (0.954-1.029) | 0.631 |  |  |
| **BMI** |  | 0.264 |  |  |
| **<25** | 1 |  |  |  |
| **≥25** | 1.527 (0.726-3.209) |  |  |  |
| **Pre-to-post ALC ratio** | 1.955 (1.414-2.703) | <0.001 | 1.577 (1.087-2.290) | 0.017 |
| **ER** |  | 0.003 |  | 0.020 |
| Negative | 1 |  | 1 |  |
| Positive | 0.328 (0.156-0.689) |  | 0.376 (0.165-0.858) |  |
| **PR** |  | 0.011 |  | 0.733 |
| Negative | 1 |  | 1 |  |
| Positive | 0.410 (0.207-0.813) |  | 0.856 (0.349-2.096) |  |
| **HER2** |  | 0.046 |  | 0.023 |
| Negative | 1 |  | 1 |  |
| Positive | 2.016 (1.012-4.013) |  | 2.272 (1.119-4.616) |  |
| **HG** |  | 0.005 |  | 0.069 |
| Ⅰ,Ⅱ | 1 |  | 1 |  |
| Ⅲ | 2.942 (1.390-6.228) |  | 2.186 (0.940-5.086) |  |
| **Stage** |  | <0.001 |  | <0.001 |
| I | 1 |  | 1 |  |
| II | 7.528 (3.606-15.719) |  | 5.402 (2.451-11.906) |  |
| III | 18.891 (5.514-64.719) |  | 13.442 (3.847-46.975) |  |

HR, hazard ratio; 95%CIs, 95% confidence intervals; BMI, body mass index; ALC, absolute lymphocyte count; ER, estrogen receptor; PR, progesterone receptor; HER2, human epidermal growth factor receptor 2; HG, histologic grade

**Supplementary Table 2. Clinical characteristics according to pre-to-post ALC ratio**

|  | **ALC ratio>2.4, n=112 (%)** | **ALC ratio≤2.4, n=419 (%)** | ***P* value** |
| --- | --- | --- | --- |
| **Age (year, continuous)** | 52.21±9.94 | 51.40±9.78 | 0.433 |
| **BMI (kg/m^2^, continuous)** | 23.74±3.23 | 23.06±3.17 | 0.046 |
| **Pre-ALC (cells/μL)** | 2169.51±583.11 | 1786.97±541.63 | <0.001 |
| **Post-ALC (cells/μL)** | 718.03±228.85 | 1100.28±342.47 | <0.001 |
| **ER** |  |  | 0.071 |
| **Positive** | 91 (81.3) | 368 (87.8) |  |
| **Negative** | 21 (18.8) | 51 (12.2) |  |
| **PR** |  |  | 0.171 |
| **Positive** | 72 (64.3) | 296 (70.6) |  |
| **Negative** | 40 (35.7) | 121 (28.9) |  |
| **Missing** | 0 | 2 (0.5) |  |
| **HER2** |  |  | 0.166 |
| **Positive** | 29 (25.9) | 137 (32.7) |  |
| **Negative** | 82 (73.2) | 278 (66.3) |  |
| **Missing** | 1 (0.9) | 4 (1.0) |  |
| **HG** |  |  | 0.105 |
| **Ⅰ,Ⅱ** | 87 (77.7) | 347 (82.8) |  |
| **Ⅲ** | 18 (16.1) | 44 (10.5) |  |
| **Missing** | 7 (6.3) | 28 (6.7) |  |
| **Subtype** |  |  | 0.029 |
| **Luminal/HER2(-)** | 67 (59.8) | 251 (59.9) |  |
| **HER2 (+)** | 29 (25.9) | 136 (32.5) |  |
| **TNBC** | 15 (13.4) | 26 (6.2) |  |
| **Missing** | 1 (0.9) | 6 (1.4) |  |
| **Stage** |  |  | <0.001 |
| **I** | 85 (75.9) | 379 (90.5) |  |
| **II** | 22 (19.6) | 33 (7.9) |  |
| **III** | 3 (2.7) | 3 (0.7) |  |
| **Missing** | 2 (1.8) | 4 (1.0) |  |
| **Tumor size** |  |  | 0.309 |
| **≤2cm** | 102 (91.1) | 394 (94.0) |  |
| **>2cm** | 9 (8.0) | 23 (5.5) |  |
| **Missing** | 1 (0.9) | 2 (0.5) |  |
| **Node metastasis** |  |  | <0.001 |
| **No** | 92 (82.1) | 403 (96.2) |  |
| **Yes** | 19 (17.0) | 15 (3.6) |  |
| **Missing** | 1 (0.9) | 1 (0.2) |  |
| **Endocrine treatment** |  |  | 0.059 |
| **Done** | 95 (84.8) | 381 (90.9) |  |
| **Not done** | 17 (15.2) | 38 (9.1) |  |
| **RNI** |  |  | <0.001 |
| **Done** | 42 (37.5) | 37 (8.8) |  |
| **Not Done** | 59 (52.7) | 341 (81.4) |  |
| **Missing** | 11 (9.8) | 41 (9.8) |  |
| **RT dose (cGy, continuous)** | 5939.45±100.90 | 5907.33±284.66 | 0.060 |

ALC, absolute lymphocyte count; BMI, body mass index; ER, estrogen receptor; PR, progesterone receptor; HER2, human epidermal growth factor receptor 2; HG, histologic grade; TNBC, triple negative breast cancer; RNI, regional nodal irradiation; RT, radiotherapy

**Supplementary Table 3. Hazard ratios (HRs) and 95% confidential intervals (CIs) for recurrence-free survival (RFS)**

|  | **Univariate analysis** | | **Multivariate analysis** | |
| --- | --- | --- | --- | --- |
|  | **HR (95% CIs)** | ***P* value** | **HR (95% CIs)** | ***P* value** |
| **Age** | 0.991 (0.954-1.029) | 0.631 |  |  |
| **BMI** | 1.055 (0.957-1.162) | 0.284 |  |  |
| **Pre-to-post ALC ratio** |  | 0.001 |  | 0.020 |
| **≤2.4** | 1 |  | 1 |  |
| **>2.4** | 3.375 (1.701-6.698) |  | 2.380 (1.146-4.942) |  |
| **ER** |  | 0.003 |  | 0.015 |
| Negative | 1 |  | 1 |  |
| Positive | 0.328 (0.156-0.689) |  | 0.256 (0.086-0.764) |  |
| **PR** |  | 0.011 |  | 0.456 |
| Negative | 1 |  | 1 |  |
| Positive | 0.410 (0.207-0.813) |  | 0.718 (0.301-1.714) |  |
| **HER2** |  | 0.046 |  | 0.018 |
| Negative | 1 |  | 1 |  |
| Positive | 2.016 (1.012-4.013) |  | 2.346 (1.158-4.758) |  |
| **Subtype** |  |  |  |  |
| Luminal/HER2(-) | 1 |  | 1 |  |
| HER2 (+) | 2.496 (1.184-5.260) | 0.016 | 1.959 (0.883-4.346) | 0.098 |
| TNBC | 3.167 (1.129-8.887) | 0.029 | 0.497 (0.113-2.182) | 0.354 |
| **HG** |  | 0.005 |  | 0.057 |
| Ⅰ,Ⅱ | 1 |  | 1 |  |
| Ⅲ | 2.942 (1.390-6.228) |  | 2.266 (0.977-5.252) |  |
| **Stage** |  |  |  |  |
| I | 1 |  | 1 |  |
| II | 7.528 (3.606-15.719) | <0.001 | 5.587 (2.559-12.200) | <0.001 |
| III | 18.891 (5.514-64.719) | <0.001 | 12.994 (3.675-45.941) | <0.001 |

HR, hazard ratio; 95%CIs, 95% confidence intervals; BMI, body mass index; ALC, absolute lymphocyte count; ER, estrogen receptor; PR, progesterone receptor; HER2, human epidermal growth factor receptor 2; HG, histologic grade
